# Deciphering Small Noncoding RNAs during the Transition from Dormant Embryo to Germinated Embryo in Larches (*Larix leptolepis*)

**DOI:** 10.1371/journal.pone.0081452

**Published:** 2013-12-10

**Authors:** Junhong Zhang, Shougong Zhang, Suying Han, Xinmin Li, Zaikang Tong, Liwang Qi

**Affiliations:** 1 Nurturing Station for the State Key Laboratory of Subtropical Silviculture, Zhejiang Agriculture and Forestry University, Lin’an, Hangzhou, Zhejiang, P.R. China; 2 Laboratory of Cell Biology, Research Institute of Forestry, Chinese Academy of Forestry, Beijing, P.R. China; 3 Research Institute of Forest Ecology, Environment and Protection, Chinese Academy of Forestry, Beijing, P.R. China; 4 Department of Pathology and Laboratory Medicine, University of California Los Angeles, Los Angeles, California, United States of America; Oregon State University, United States of America

## Abstract

Small RNAs (sRNAs), as a key component of molecular biology, play essential roles in plant development, hormone signaling, and stress response. However, little is known about the relationships among sRNAs, hormone signaling, and dormancy regulation in gymnosperm embryos. To investigate the roles of sRNAs in embryo dormancy maintenance and release in *Larix leptolepis*, we deciphered the endogenous “sRNAome” in dormant and germinated embryos. High-throughput sequencing of sRNA libraries showed that dormant embryos exhibited a length bias toward 24-nt while germinated embryos showed a bias toward 21-nt lengths. This might be associated with distinct levels of RNA-dependent RNA polymerase2 (*RDR2*) and/or *RDR6*, which is regulated by hormones. Proportions of miRNAs to nonredundant and redundant sRNAs were higher in germinated embryos than in dormant embryos, while the ratio of unknown sRNAs was higher in dormant embryos than in germinated embryos. We identified a total of 160 conserved miRNAs from 38 families, 3 novel miRNAs, and 16 plausible miRNA candidates, of which many were upregulated in germinated embryos relative to dormant embryos. These findings indicate that larches and possibly other gymnosperms have complex mechanisms of gene regulation involving miRNAs and other sRNAs operating transcriptionally and posttranscriptionally during embryo dormancy and germination. We propose that abscisic acid modulates embryo dormancy and germination at least in part through regulation of the expression level of sRNA-biogenesis genes, thus changing the sRNA components.

## Introduction

Japanese larch (*Larix leptolepis*), a uniquely suitable Pinaceae for the experimental study of gymnosperms, is one of the most important forestry trees in northern China, Russia, Europe, and Japan [1], [2]. The germination of the seed embryo is a critical step in the production of larches. Due to their inaccessibility along with difficulties associated with gymnosperm seed embryos, we used somatic embryogenesis as an experimental system to gain information about morphological and molecular changes that take place during embryo dormancy and germination. Somatic embryogenesis is defined as a process in which a bipolar structure resembling a zygotic embryo is used as a model for studying the regulation of embryo development [3].

Seed dormancy and germination are regulated by developmental and environmental cues [4], [5]. Maturation of seed embryos leads to a concomitant gradual decrease of metabolism as water is lost from the seed tissue and the embryo passes into a metabolically inactive, or quiescent, state [6]. Previous studies showed that the balance between abscisic acid (ABA) and gibberellin (GA) is important for determining the dormancy status of seed embryos [7]. ABA is abundant in dormant seeds and generally decreases when dormancy is released [8], whereas some GAs increase during the process of germination [9]. ABA metabolism and signaling potentially act as the node for hormone cross talk [10]. Recent progress in basic research has advanced our understanding of the mechanisms of seed dormancy and germination in model plant species, but little is known regarding the mechanisms underlying the complex regulation of seed dormancy and germination by transcriptional and posttranscriptional controls, especially for gymnosperms.

Mounting evidence has revealed that small RNAs (sRNAs), exemplified by microRNAs (miRNAs), play pivotal roles in each major stage of plant development, mediating the transition from one developmental stage to the next [11], [12]. RNA silencing directed by sRNAs is a highly conserved regulatory mechanism known to be involved in diverse processes, such as development, hormone signaling, antiviral defense, genome maintenance, and stress response [13], [14]. Ever since the ground-breaking discovery of sRNAs [15], [16], the goal of many laboratories has been to decipher the endogenous “sRNAome” of living organisms with the aim to understand its biogenesis and function. sRNAs mainly include miRNAs and several forms of small interfering RNAs (siRNAs), including *trans*-acting siRNAs (ta-siRNAs), natural antisense siRNAs (nat-siRNAs), and repeat-associated siRNAs (ra-siRNAs). These sRNAs are usually 20- to 24-nucleotides (nt) in length and are found in nearly all eukaryotes [11], [17]. In plants, the pool of sRNAs is complex and dynamic, consisting primarily of many low-abundant siRNAs and a small number of highly expressed miRNAs [13], [14].

*Arabidopsis* has four Dicer-like (*DCL*) proteins with distinct, hierarchical, and overlapping functions in sRNA biogenesis. Most miRNAs are processed by *DCL1* enzymes [18], although a few evolutionarily young miRNAs are generated by *DCL4* [19]. *DCL3* produces 24-nt ra-siRNAs at heterochromatic loci [20] and is conserved in both angiosperms and mosses [21], [22]. Several previous studies have indicated the absence of *DCL3* in conifers by expressed sequence tag (EST) searching; thus, conifers fail to show appreciable amounts of 24-nt sRNAs [23], [24], [25]. Unexpectedly, two recent reports have documented homologs of *DCL3* in two gymnosperm species, *Cunninghamia lanceolata* and *Larix principis-rupprechtii* [26], [27]. RNA-dependent RNA polymerase (*RDR*) is known to be responsible for the synthesis of double-stranded RNA on single-stranded RNA substrates. *Arabidopsis* has six *RDR* genes, of which *RDR2* is a crucial factor in the biogenesis of ra-siRNAs, representing over 90% of these siRNAs [28], [29]. *RDR*6 has been implicated in the biogenesis of siRNAs from plant viruses and transgenes and also acts in the biogenesis of ta-siRNAs [30].

Recent evidence has implicated hormone signaling in the regulation of sRNAs by affecting the abundance of sRNA pathway genes. *OsRDR6*-dependent siRNA generation is significantly upregulated by ABA, while four other *RDRs*, including *RDR2*, are not regulated by ABA in *Oryza sativa* [31]. miR168 controls *AGO1* homeostasis during ABA treatment and abiotic stress responses in *Arabidopsis thaliana* [32]. GA has been shown to regulate miR159 levels during anther development [33]. Several sRNA target genes have roles in auxin signaling, such as miR160, miR393, miR167, and tasiR-*ARF*s [34]. Furthermore, miRNAs regulated by hormones are associated with seed germination. ABA induction of miR159 controls *MYB33* and *MYB101*, and overexpression of miR159 or mutations in *MYB33* and *MYB101* lead to ABA hyposensitivity [35]. *ARF10*, *ARF16*, and *ARF17* targeted by miR160 have roles in auxin signaling, which is critical for seed germination and post-germination stages [36], [37]. Auxin-ABA cross talk is present in imbibed seeds and the downregulation of *ARF10* by miR160 is essential for the auxin–ABA cross talk during germination [38]. Compared to the information available for the roles of miRNA in embryo dormancy and germination in angiosperms, little is known in gymnosperms, in which the anatomy and biology of the cell and the molecular mechanisms used during embryogenesis differ significantly from those in angiosperms [39], [40].

In this study, somatic embryos were used to decipher the dynamic sRNAome during the transition from dormant embryos into germinated embryos in *L. leptolepis*. Our results showed that the length distribution and components of sRNAs change dramatically between these two classes of embryos, which may be attributable to distinct *RDR2* and *RDR6* expression regulated by hormone content. In total, 160 conserved miRNAs from 38 families, 3 novel miRNAs, and 16 plausible miRNA candidates were identified. Most miRNAs were upregulated in germinated embryos relative to dormant embryos and their targets showed the opposite expression pattern.

## Results

### The Morphology of the Dormant and Germinated Larch Embryos

**Figure 1:**
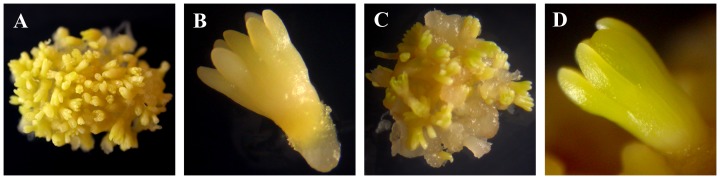
The morphology of dormant and germinated embryos in larch. (A and B) The embryos entered into dormant or quiescent status when the embryos were faint yellow. (C and D) The embryo with larger size turned yellow–green, especially the cotyledons, after culture for 12 days on non-ABA medium.

After mature somatic embryos were grown for 12 days on ABA-containing medium, the embryos entered into a dormant or quiescent phase and became faint yellow. However, the germinated embryos turned yellow–green, especially the cotyledons, after culture for 12 days on non-ABA medium (Figure 1). In addition, the germinated embryos were larger than these of dormant embryos, which might have been due to increased cell elongation and cell division in dormancy released embryos. Considerable elongation of the embryos, exemplified by the cotyledons, was due mainly to cell division in the cotyledons.

### Hormone Content of the Dormant and Germinated Embryos

**Figure 2:**
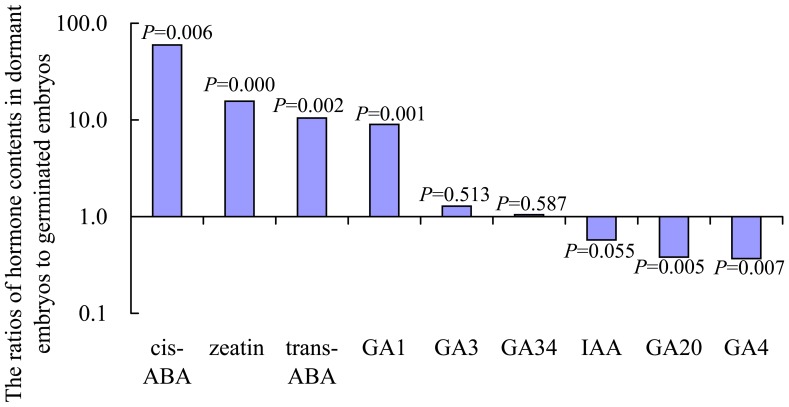
The hormone contents were compared between dormant embryos treated with ABA and germinated embryos with no ABA after embryo maturation. Presented values are the mean (±SE) content of hormones of three independent samples per treatment.

The hormone content differed significantly between dormant embryos and germinated embryos (Figure 2). The level of *cis*/*trans*-ABA was significantly higher in dormant embryos than in germinated embryos. Thus, the content of three hormones, IAA, GA_4_, and GA_20_, was higher in germinated embryos than in dormant embryos. Unexpectedly, GA_1_, GA_3_, GA_34_, and zeatin were more abundant in dormant embryos than in germinated embryos. However, the ratio of ABA to GAs was much lower in germinated embryos than in dormant embryos.

### High-throughput Sequencing Reveals a Dynamic Change in sRNAs from Dormant Embryo to Germination

In total, 16,514,593 and 16,415,719 raw reads were obtained from the dormant and germinated embryo sRNA libraries, and the accession number was GSM1214805 and GSM1193109, respectively. After removal of nonsense reads, we obtained clean reads with lengths of 18–30 nt, representing 16,342,531 (98.96%) and 16,123,435 (98.22%) sequences and 5,644,091 and 2,880,823 nonredundant sequences, respectively. These high-quality sRNAs were used for further analysis.

**Figure 3:**
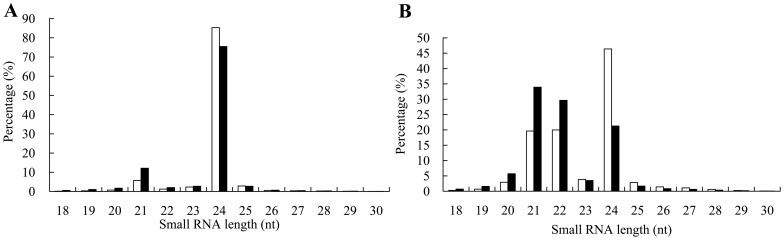
Length distribution of sRNA sequences in larch embryos. (A) Frequencies are expressed as the percentage of the total nonredundant sequences for dormant embryos (white bars) and germinated embryos (black bars). (B) Frequencies are expressed as the percentage of the total redundant sequences for dormant embryos (white bars) and germinated embryos (black bars).

Both batches contained a similar size distribution of nonredundant sRNAs, with 24-nt representing the largest class in batch 1 (85%, white bars) and batch 2 (75%, black bars in Figure 3A). Another minor peak was at 21-nt, accounting for 5.7% in batch 1 and 12.1% in batch 2. However, the overall distribution of the redundant sRNAs was strikingly different in the two libraries (Figure 3B), with one major peak at 24-nt and another minor peak at 21-nt or 22-nt in dormant embryos (white bars). Once the embryo germinated, the proportion of 24-nt sRNAs decreased and the 21-nt population increased to a major peak (black bars). Furthermore, the 24-nt class of sRNAs exhibited the lowest redundancy in both libraries, with an average frequency of 1.57 and 1.58 per nonredundant sequence, while the 22-nt length showed the highest redundancy, with an average frequency of 46.36 and 82.15 per nonredundant sequence in dormant embryos and germinated embryos, respectively.

### Deciphering the Components of sRNAs in Dormant and Germinated Embryos

**Figure 4:**
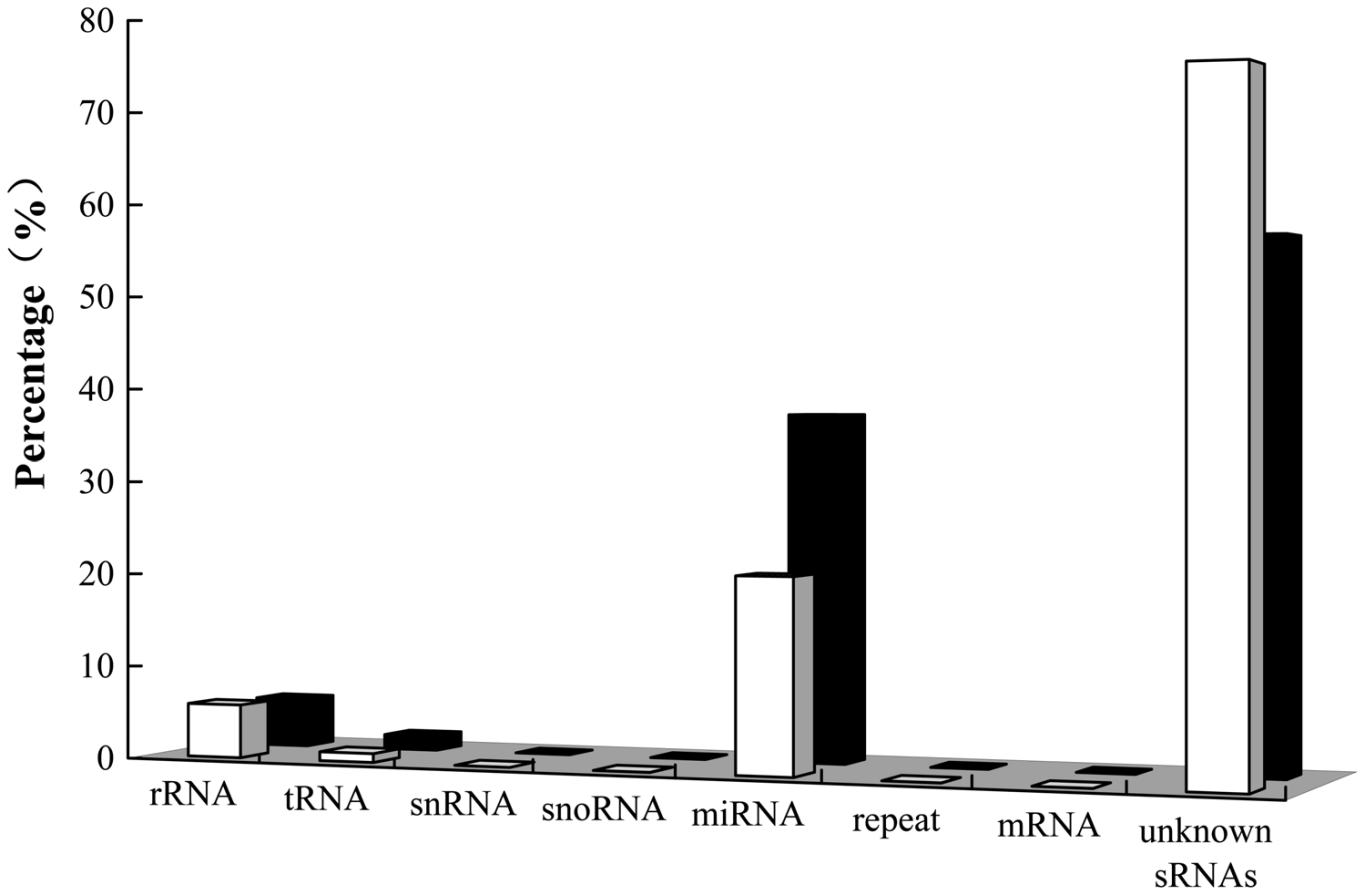
Distribution of sRNA annotation categories in dormant embryos (white bars) and germinated embryos (black bars). Sequences that matched to known RNAs accounted for small fractions, 27.40% and 44.05% of the redundant sequences in the dormant and germinated libraries, respectively. Thus, an overrepresented part of the nonredundant sRNAs was composed of unknown sRNAs.

Sequences were aligned to known RNAs in Rfam 11.0 [41], GenBank, Repeat–repbase [42], miRBase 19.0 (http://www.mirbase.org) [43], and larch miRNAs [44], including rRNAs, tRNAs, snRNAs, snoRNAs, miRNAs, ra-siRNAs, and mRNA fragments. Sequences that matched to known RNAs accounted for small fractions, 1.10% and 2.29%, respectively, of the nonredundant sequences and 27.40% and 44.05% of the redundant sequences in the dormant and germinated libraries, respectively. Thus, an overrepresented part of the nonredundant sRNAs was composed of unknown sRNAs (Figure 4). Note that the proportion of miRNAs in germinated embryos (37.25%) was higher than in dormant embryos (20.70%); nonredundant miRNAs accounted for 0.42% in germinated embryos and 0.20% in dormant embryos. Unexpectedly, a relatively small amount of raw reads, 5.72% in dormant embryos and 5.23% in germinated embryos, matched rRNAs.

### Expression Patterns of *DCL1*, *DCL3*, *RDR2*, and *RDR6* in Dormant Embryos and Germinated Embryos

**Figure 5:**
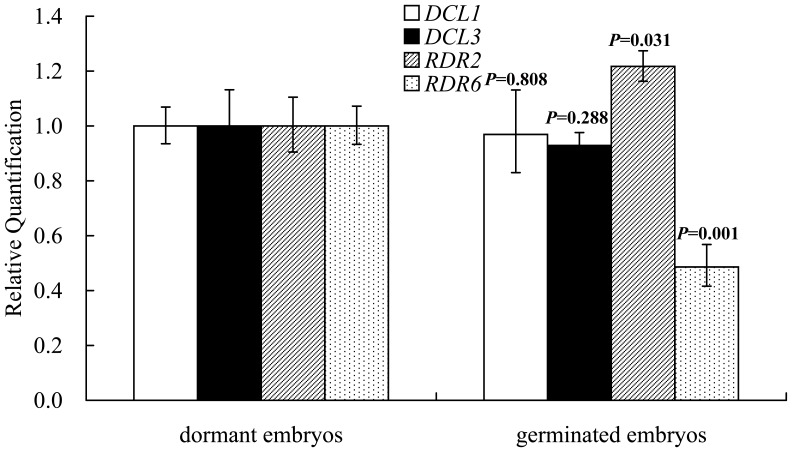
qRT-PCR analysis of the relative expression of *DCL1* (white bars), *DCL3* (black bars), *RDR2* (striped bars), and *RDR6* (dotted bars) in dormant embryos and germinated embryos. All expression levels were normalized to that of EF-1α and then normalized by comparison to the level of dormant embryos, which was set at 1.0. The experiments were repeated three times and error bars represent the SD. The abundance difference of genes between two classes of embryos was evaluated by a *t*-test using SPSS software (SPSS Inc., Chicago, IL).

To determine the expression changes of *DCL1*, *DCL3*, *RDR2*, and *RDR6* between dormant embryos and germinated embryos, we assayed the gene expression levels of embryos when cultured in ABA or non-ABA medium using qRT-PCR (Figure 5). Unexpectedly, *DCL1* and *DCL3* were not differentially expressed between dormant and germinated embryos. However, the expression level of *RDR2* was significantly higher in germinated embryos relative to dormant embryos, while *RDR6* showed the opposite expression pattern between these two embryo classes, which was consistent with the low level of ABA.

### miRNA Identification and Profiling: Dormant Embryos and Germinated Embryos

Currently, miRNAs from 18 broadly conserved families, including 81 members, have been identified, as well as 20 families that were species-specific or restricted to certain plant families, exemplified by 37 miRNAs from 9 families that appear to be specific to gymnosperms or conifers (Table 1). We followed a homolog-based approach to search for already known miRNAs in our two sRNA libraries using miRBase (release 19.0) as a reference set. The miRNA abundance among the families was notably variable in dormant embryos and germinated embryos. For example, miR950, miR165/166, and miR156/157 were most highly expressed and were detected 2,232,537, 481,738, and 217,941 times in dormant embryos and 3,538,778, 1,064,904, and 522,272 times in germinated embryos, respectively. However, miR160, miR393, and miR827 were detected two, three, and three times in dormant embryos and six, nine, and seven times in germinated embryos, respectively.

**Table 1:** Identification of known miRNAs in larch.

18 highly conserved miRNA families				
Family Name (number of members)	Conserved species (number of members)			
	Number	ath	osa	others
miR156/157 (8)	41	11	12	pta (2)
miR159/319/4414 (16)	32	6	8	pta (4)
miR160 (1)	33	3	6	pab (2)
miR162 (2)	25	2	2	ptc (3)
miR164 (5)	26	3	6	ptc (6)
miR165/166 (16)	35	9	14	pab (2)
miR167 (7)	32	4	10	ptc (8)
miR168 (3)	26	2	2	ptc (2)
miR169 (3)	27	14	17	ptc (32)
miR171 (2)	36	3	9	pta (1)
miR172 (4)	30	5	4	ptc (9)
miR390 (2)	23	2	1	pta (1)
miR393 (1)	22	2	2	ptc (4)
miR396 (3)	35	2	9	pab (3)
miR397 (2)	21	2	2	pab (1)
miR398 (2)	25	3	2	pta (1)
miR399 (2)	29	6	11	ptc (12)
miR408 (2)	28	1	1	pta (1)
**20 known families showed partial conservation with other plants**				
**Family Name (number of members)**	**Conserved species (number of members)**			
	**Number**	**pta**	**pab**	**others**
miR894 (8)	1	/	/	ppt (1)
miR1083 (1)	1	/	/	smo (1)
miR5059 (5)	1	/	/	bdi (1)
miR5139 (4)	1	/	/	rgi (1)
miR482 (4)	22	4	4	ptc (3)
miR528 (2)	5	/	/	osa (1)
miR529 (3)	9	/	/	ppt (7)
miR535 (5)	9	/	1	ppt (4)
miR536 (1)	2	/	/	ppt (5)
miR827 (1)	12	/	/	ath (1)
miR2118 (2)	9	/	/	osa (18)
miR947 (1), miR950 (12), miR951 (4), miR1311 (5)	3	1	1	pde (1)
miR946 (6), miR1312 (2), miR1313 (2),miR1314 (2), miR3701 (3)	2	1	/	pde (1)

ath: *Arabidopsis thaliana*; osa: *Oryza sativa*; ptc: *Populus trichocarpa*; pta: *Pinus taeda*; pab: *Picea abies*; ppt: *Physcomitrella patens*; smo: *Selaginella moellendorffii*; rgi: *Rehmannia glutinosa*; bdi: *Brachypodium distachyon*; pde: *Pinus densata*.

To uncover additional larch-specific miRNA candidates within our sequence data set, sequences were aligned against the *L. leptolepis* ESTs. Nineteen ESTs containing 19 potential miRNA precursors were selected for further analysis. Of these, three mature miRNA sequences with their corresponding miRNA* sequences were identified as novel miRNAs, together with 16 candidate miRNAs (Table 2).

**Table 2:** Isolation and identification of novel miRNAs in larch embryo.

Name	Sequence(5′-3′)	Nucleotide(length)	Read(Dormant embryo)	Read(Germinated embryo)	Containing EST
llemiR-1	UGCCGUGGUUCGGAGCGAUCGA	22	11005	15905	JR179967
llemiR-1*	AUCCUCCCAACCAAGGCAACC	21	2	7	JR179967
llemiR-2	UGACCAGUCCUUCUGCGAUCCA	22	148	162	JR164575
llemiR-2*	AAUUGCAGAAGGGCUGGUUAGC	22	789	1007	JR164575
llemiR-3	UCAAGUGUUUCUGGACUCACC	21	5	0	JR164003
llemiR-3*	UGAGUCCAGACACACUUCGGC	21	1	1	JR164003
llemiR-4	UUUGAUAGAUCCGAGGUUAAG	21	70	64	PEMSG842X^a^
llemiR-5	UGCAAAUGGUGUUUGCGUCGU	21	16	26	JR170741
llemiR-6	UCCAUGACUUUCCAGAGGGGU	21	336	298	JR184550
llemiR-7	UCAUUCCAGUUAUCGUUCUCC	22	50	74	JR185282
llemiR-8	UCUGCCUGGUACCUUGACGUA	21	98	124	JR174289
llemiR-9	UCGCAGGUGAGAUGACGCCGGC	22	52	88	JR160786
llemiR-10	UGAGCUCUUGGAAGUGUUGGA	21	205	290	PEMSJJR7E^a^
llemiR-11	GCCGUGACCGUGGCGAUCGUGG	22	9	7	JR167874
llemiR-12	UACACCUCAAGAAAUUGGAUCCCU	24	4	1	JR176014
llemiR-13	UGAUUCAGGCAUGGAGGAGGACUA	24	10	1	JR179449
llemiR-14	UCUUUCUGAGGCAUGUAUGGGCAU	24	6	1	JR181565
llemiR-15	UCAGUGAGCUUAGGGUACGUUGG	23	0	4	JR184842
llemiR-16	UCGGAAUGCUGGAGGAGGCAA	21	1	6	JR190670
llemiR-17	UGAGUCCAGACACACUUCGGCU	22	4	3	JR164003
llemiR-18	CAACGAUCAACAGGACCACUG	21	24	16	JR195099
llemiR-19	UGUGACGGGGAUGGGAUGCU	20	0	11	JR155419

a The sequences of PEMSG842X and PEMSJJR7E containing the mature sequence of llemiR-4 and llemiR-10 were not submitted to the TSA database because their length were less than 200 nt.

**Figure 6:**
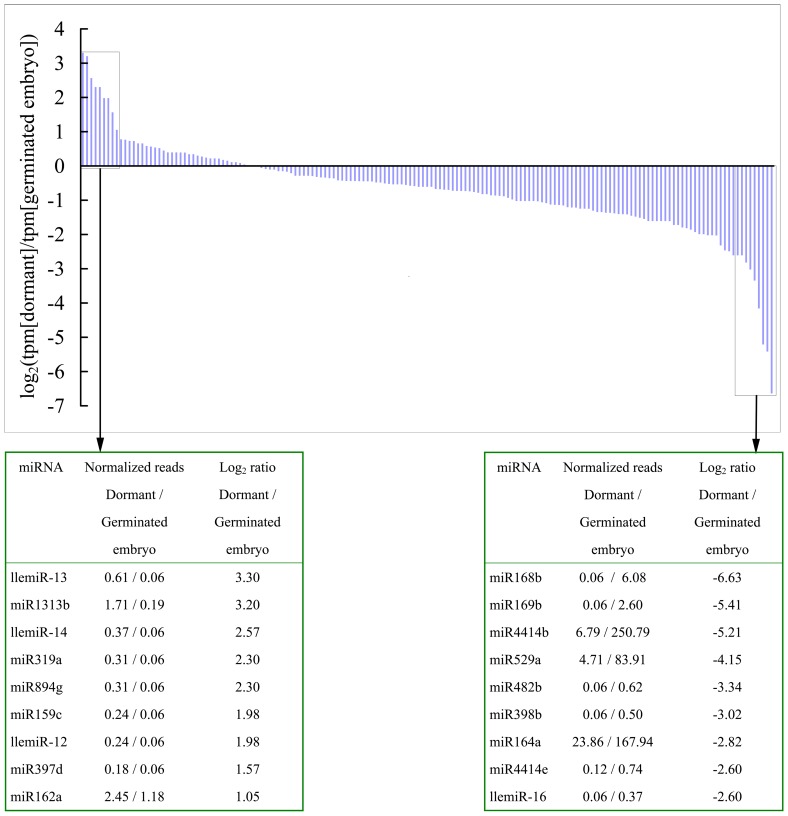
Differential expression of miRNAs in dormant and germinated larch embryos. Expression ratios (the percentages of normalized reads in dormant embryos divided by the percentages of normalized reads in germinated embryos) are shown for all miRNAs that were detected in both the dormant embryo and germinated embryo data sets. Specific data pertaining to the nine most differentially expressed miRNAs at both ends of the spectrum are displayed in the inset, and reads were normalized to tags per million as described in the Methods.

The normalized abundance of individual miRNAs and calculation of log_2_(tpm[dormant embryos]/tpm[germinated embryos]) can be used to compare the relative abundance of miRNAs between the two stages of larch embryos, as reported previously for *Populus* [45]. The miRNAs for which log_2_(tpm[dormant embryos]/tpm[germinated embryos]) were greater than 1 or less than –1 were considered to be up- or downregulated, respectively. In total, 163 miRNAs or miRNA candidates were included in the comparison. The results showed that llemiR-13, miR1313b, llemiR-14, miR319a, miR894g, miR159c, llemiR-12, miR397d, and miR162a were upregulated in dormant embryos, whereas 61 miRNAs, including miR168b, miR169b, miR4414b, and miR529a, were upregulated in germinated embryos (Figure 6 and Table S1). In addition, some miRNAs were expressed in a single library because no corresponding reads were found in the other library; therefore, these could not be assigned a fold-overexpression value. Four miRNAs were represented only in dormant embryos by three or more reads, while 11 miRNAs were detected only in germinated embryos (Table S1). The results of qRT-PCR validated the sequencing data, albeit with smaller fold-changes between dormant embryos and germinated embryos (Figure S1).

### Differential Expression Patterns of miRNAs and Their Target Genes between Dormant Embryos and Germinated Embryos

**Figure 7:**
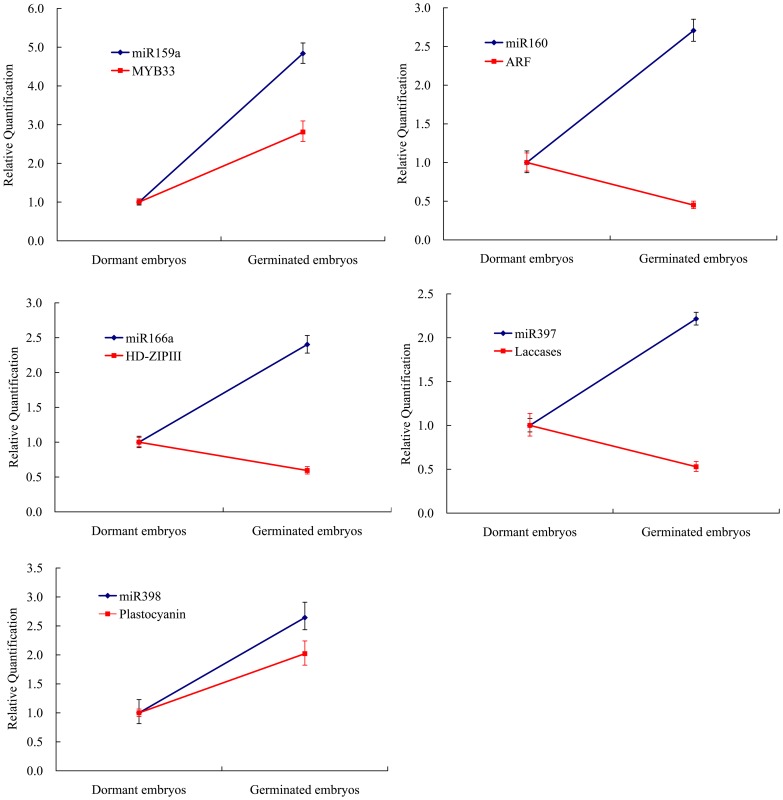
qRT-PCR-derived expression analysis of five miRNAs and their target genes in dormant embryos and germinated embryos. All expression levels were normalized to that of EF-1α and then normalized by comparison to the level of dormant embryos, which was set at 1.0. The experiments were repeated three times and error bars represent the SD.

Referring to the known function of conserved miRNAs, five miRNAs and their target genes were selected as candidate miRNAs related to embryo dormancy and germination. The expression levels of all detected miRNAs were higher in germinated embryos than in dormant embryos, but with distinct fold changes (Figure 7), which was consistent with the results from the high-throughput sequencing of sRNA libraries, except for miR397. Accordingly, the abundance for three target genes was lower in germinated embryos than in dormant embryos, except for *MYB33* and *plastocyanin*, targeted by miR159 and miR398, respectively. The abundance of *MYB33* and *plastocyanin* was higher in germinated embryos than in dormant embryos by 2.81- and 2.02-fold, respectively.

## Discussion

### Differential Hormone Content between Dormant Embryos and Germinated Embryos

Many reasons exist as to why viable embryos and/or seeds do not germinate, and a block in the completion of germination is usually a consequence of multiple events. We have seen a tremendous advance in understanding of hormonal regulation in embryo germination, exemplified by ABA promotion of somatic embryo maturation and entry into dormancy [46], with ABA metabolism and signaling potentially acting as the node for hormone cross talk [10]. Previous studies showed that the balance between ABA and GA is important for determining the dormancy status of seed embryos [7]. Because the actions of hormones are mutually interactive, the consequences of a change in content of a single hormone may be quite different from one state to another in the target tissue [47]. Therefore, simultaneous quantification of multiple hormones from the same plant material is a useful methodology to examine the overall picture of hormone balance [48].

In our study, the content of IAA was not significantly lower in dormant embryos than in germinated embryos, which is consistent with a previous study reporting that IAA content is relatively low in mature embryos [49]. The abundance of *cis*/*trans*-ABA was significantly higher in dormant embryos than in germinated embryos, which is consistent with ABA abundance in dormant seeds that generally decreases when seed dormancy is released [8]. Unexpectedly, all detected GAs, except for GA4, were abundant in dormant embryos and at lower levels in germinated embryos. This is opposite to the result that some GAs increase during the transition to germination [9]. However, the ratio of ABA to GAs was much lower in germinated embryos than in dormant embryos, which is consistent with the result that the balance between ABA and GA is important for determining the dormancy status of embryos [7]. Zeatin was more abundant in dormant embryos than in germinated embryos in larches. A similar observation was previously made in a study by Brzobohaty et al. (1993), who observed a high content of ribosylzeatin and low level of active zeatin in dormant seeds, while active zeatin increased rapidly via decreasing zeatin conjugates. In conclusion, a decrease in the ABA content of embryos via the withdrawal of exogenous ABA in the medium alters the content of other hormones, which was supported in a study by Nambara et al. [10], who suggested that ABA metabolism and signaling act as the node for hormone cross talk.

### Diverse sRNAs Participate in Embryo Dormancy and Germination

Previous studies indicate that conifers fail to express appreciable amounts of 24-nt sRNAs [23], [24], [25]. However, two recent reports have identified *DCL3* homologs and have observed diverse 24-nt sRNAs in two gymnosperm species [26], [27]. In this study, the 24-nt sRNAs were the most diverse, exemplified by more than three-quarters of nonredundant sequences being 24-nt in size and accounting for 85% of the nonredundant sRNAs in dormant embryos. A similar phenomenon is observed in the olive (*Olea europaea*) in which unique 24-nt sequences account for 80% of the “sRNAome” [50]. However, 60% of the nonredundant sRNAs are 24-nt long in *Arabidopsis* [51] with 22% in *O. sativa* [23]. This implies that woody perennials, including gymnosperms and angiosperms, contain a more abundant diversity of 24-nt sRNA than annual herbaceous plants, although the latter may contain a higher proportion of 24-nt redundant sRNAs. Thus, annual herbaceous plants show significantly higher DNA methylation levels than woody perennials [52].

The overall distribution of the redundant sRNAs differed strikingly in the two libraries, with one major peak at 24-nt and another minor peak at 21-nt or 22-nt in dormant embryos, while in germinating embryos, the proportion of 24-nt sRNAs decreased and the 21-nt population increased to a major peak. This was consistent with our previous study reporting that 24-nt sRNAs predominate during embryogenesis, while the distribution patterns change upon embryo germination [27]. Several studies showed that the 24-nt fraction is dominated by sRNA derived from genomic repeats, transposons, and intergenic regions. Thus, most of these sRNAs mainly act as heterochromatin siRNAs in *O. sativa* [23], *A. thaliana* [53], and *Gossypium hirsutum* [54]. Therefore, we speculate that tremendous alteration of epigenetic regulation exists between dormant embryos and germinated embryos.

Unexpectedly, a relatively small amount of raw reads, 5.72% in dormant embryos and 5.23% in germinated embryos, matched rRNAs, which suggested that a large proportion of rRNAs are “dormant” in embryos. A similar phenomenon is observed in cotton (*G. hirsutum*) ovules, in which the ratio of rRNA fragments to total reads in ovules with fiber cell initials and young fiber-bearing ovules is only 6.37% and 7.03%, respectively. However, the corresponding ratios are much higher in leaves and immature ovules, accounting for 53.83% and 29.56%, respectively [54]. This implies that rRNA degradation is highly regulated, representing a high proportion of rRNAs that are degraded in leaves and immature ovules. Alternatively, the rRNA genes in leaves and immature ovules may be subjected to silencing or nucleolar dominance via RNA-mediated pathways [55]. Moreover, the proportion of miRNAs in germinated embryos (37.25%) was higher than in dormant embryos (20.70%), which implies that miRNAs participate in embryo germination. However, nonredundant miRNA sequences accounted for 0.20% in dormant embryos and 0.42% in germinated embryos. This was consistent with the results that plant sRNAs consist primarily of many low-abundant siRNAs with a small number of highly expressed 21-nt sequences and most of the latter are miRNAs [13], [14].

### The Response of sRNA Pathway Genes to Hormones

In this study, the expression level of *RDR2* was significantly higher in germinated embryos relative to dormant embryos, while *RDR6* had the opposite expression pattern. Furthermore, we observed that a decreasing level of ABA led to a concomitant decrease in *RDR6*. This was similar to *OsRDR6*, which is positively regulated by ABA, while *OsRDR2* is not regulated by ABA [31]. Our previous study showed that *RDR2* accumulated at the lowest level in mature embryos, while it increased in seedlings that originated from somatic embryos [27]. The abundance of *RDR6* in germinated embryos was lower than that of dormant embryos, which seems to contradict sRNA composition. The possibility exists that the content of *RDR6* was sufficient to the production of siRNAs, or that those siRNAs dependent on *RDR6* may not have been the main component affecting sRNA composition, while miRNAs were the main contributor. However, the transcript abundance of *DCL1* was not significantly different between dormant embryos and germinated embryos, suggesting that *DCL1* was not affected by hormones, especially by ABA. Previous studies showed that other miRNA processing genes may be regulated by hormones, thus affecting the accumulation of miRNAs. This is exemplified by the *HYL1* gene, whose expression responds to ABA, auxin, and cytokinin [56]. In our study, this correlation of ABA content and *RDR6* accumulation was consistent with the hypothesis that *RDR6* was regulated, at least in part, by ABA, thus the expression level of 21-nt ta-siRNA was altered [31].

### Potential Roles of miRNAs in Maintaining and Releasing Embryo Dormancy

Early studies on miRNA biogenesis-related mutants implied that miRNAs are essential for seed germination [56], [57], [58]. A previous study showed that miR159 induced by ABA controls transcript levels of *MYB33* and *MYB101* during *Arabidopsis* seed germination [35]. In our study, the expression level of miR159a was higher in germinated embryos than in dormant embryos by 4.8-fold (Figure 7). Moreover, the abundance of *MYB33* was also higher in germinated embryos relative to dormant embryos. Oppositely correlated expression to what is expected of conventional repressors is increasing being seen in relation to miRNA–mRNA target pairs [59]. The phenomenon of co-expression was also observed in miR398-*plastocyanin*, in which the expression levels of both miR398 and *plastocyanin* were higher in germinated embryos than in dormant embryos. *Plastocyanin* functions as a soluble carrier transferring electrons between cytochrome b(6)f and photosystem I [60]. The expression pattern of *plastocyanin* was consistent with the phenomenon that genes involved in photosynthesis and carbon fixation are upregulated from dormant embryos compared to germinated embryos [61]. This was in accord with the morphology of germinated embryos (Figure 1) in which the whole embryo turned yellow–green, especially the cotyledons.

The expression level of miR160 was higher in germinated embryos than in dormant embryos by 2.7-fold, while the mRNA of the target gene *ARF* decreased in germinated embryos relative to dormant embryos. This was consistent with the phenomenon that negative regulation of *ARF10* by miR160 plays important roles in seed germination and post-germination [36]. miR166 levels were elevated in germinated embryos and its target gene (*HD-ZIPIII*) showed the opposite expression pattern. Several studies suggested that miR166 regulates *HD-ZIPIII* expression, mediating indeterminacy in apical and vascular meristems [62], [63]. Therefore, we speculate that miR166 and the target gene *HD-ZIPIII* participates in embryo germination. miR397 showed a similar expression pattern with miR160 and miR166 and the target gene *laccases* showed the opposite expression pattern. *Laccases* are related to lignification and thickening of the cell wall in secondary cell growth [64]. The expression pattern of miR397-*laccases* accorded with cotyledon prolongation along with cell propagation. Therefore, we inferred that miR397 has an important role in regulating the thickness of the cell wall during the transition from dormant embryos into germinated embryos by cleaving the laccase mRNA.

In this study, we deciphered the endogenous “sRNAome” in dormant and germinated embryos and found that the length distribution, sRNA components, and expression pattern of miRNAs differed distinctly between dormant embryos and germinated embryos. This may be associated with differential levels of *RDR2* and/or *RDR6*, which is regulated by hormones. These findings indicate that larches have complex mechanisms of gene regulation involving miRNAs and other sRNAs during embryo dormancy and release.

## Materials and Methods

### Plant Materials

One embryogenic cell line of Japanese larch (*Larix leptolepis*), designated D878, with a high embryo maturation capacity was used in this study. Embryogenic calluses were induced from immature embryos on induction medium, followed by subculture, and cultured on ABA-containing mature medium in a dark environment at 25±2°C. After culture for 45 days in mature medium, embryogenic calli developed into mature somatic embryos. In our study, the samples were harvested at day 57. One sample, collected after embryo maturation, continued to remain for 12 days on ABA-containing medium and another was harvested after culture for 12 days on non-ABA medium. All samples were snap-frozen in liquid nitrogen and stored in liquid nitrogen until RNA extraction.

### Measurement of the Phytohormone Content

About 0.5 g of dormant embryos and germinated embryos was ground in liquid nitrogen to fine powders and vacuum lyophilized at −20°C. For each sample, 100 mg (dry weight) of the lyophilized powders was measured for GA_1_, GA_3_, GA_4_, GA_20_, GA_34_, indole-3-acetic acid (IAA), ABA, and zeatin content [65], [66]. The measurement of phytohormones with LC-MS was conducted in the lab of Prof. Xiangning Jiang (Beijing Forestry University, PR China). The experiments were repeated three times with similar results.

### sRNA Library Construction and Bioinformatics

The extraction of total RNA, construction of the sRNA library, sequencing, and bioinformatics was conducted as described previously [44]. The abundance profile analysis through high-throughput sequencing was based on the sequence reads of each library for the dormant embryos and germinated embryos. The first step was to normalize the miRNA sequence reads in the dormant embryos and germinate embryos to tags per million (normalized expression = actual miRNA count/total count of clean reads*1,000,000). The log_2_ ratio formula was log_2_(tpm[dormant embryos]/tpm [germinated embryos]).

### Quantitative RT-PCR for miRNAs and Target Genes

sRNA was isolated using a Small RNA Isolation Kit (Biotek, Beijing, China) and cDNA was synthesized using an NCode VILO miRNA cDNA Synthesis Kit (Invitrogen, Carlsbad, CA). Quantitative reverse transcription-polymerase chain reaction (qRT-PCR) was performed using a SYBR Premix EX Taq Kit (Takara, Dalian, China) on the 7500 Real-Time PCR System (Applied Biosystems, Foster City, CA). Three biological replicates for each sample were carried out. All expression levels were normalized to 5.8S ribosomal RNA (5′-GTCTGTCTGGGCGTCGCATAA-3′). Forward primers were designed based on mature miRNA sequences (Table S2). Total RNA was isolated using a Total RNA Purification Kit (Norgen Biotek Corp., Thorold, ON, Canada) and cDNA was synthesized using a Revert Aid First Strand cDNA Synthesis Kit (Fermentas, Vilnius, Lithuania). qRT-PCR was performed as described above and all expression levels were normalized to the expression of EF-1α [67]. Primers for target genes referred to previous studies [27], [44], [68] (Table S3).

## Supporting Information

Figure S1 **Validation of the miRNA levels of dormant embryos and germinated embryos by qRT-PCR.** The results of qRT-PCR validated the sequencing data, albeit with smaller fold-changes between dormant embryos and germinated embryos.
(TIF)

Table S1 **Expression profile of miRNAs between dormant and germinated embryos.**
(DOC)

Table S2 **miRNA primers used for qRT-PCR in larch.**
(DOC)

Table S3 **Primers for qRT-PCR analysis of target genes.**
(DOC)
