# Supplementary figures and images for: Deciphering Small Noncoding RNAs during the Transition from Dormant Embryo to Germinated Embryo in Larches (Larix leptolepis)
Source: PLoS One. 2013 Dec 10;8(12):e81452. doi: 10.1371/journal.pone.0081452 (PMC3858266; doi:10.1371/journal.pone.0081452)

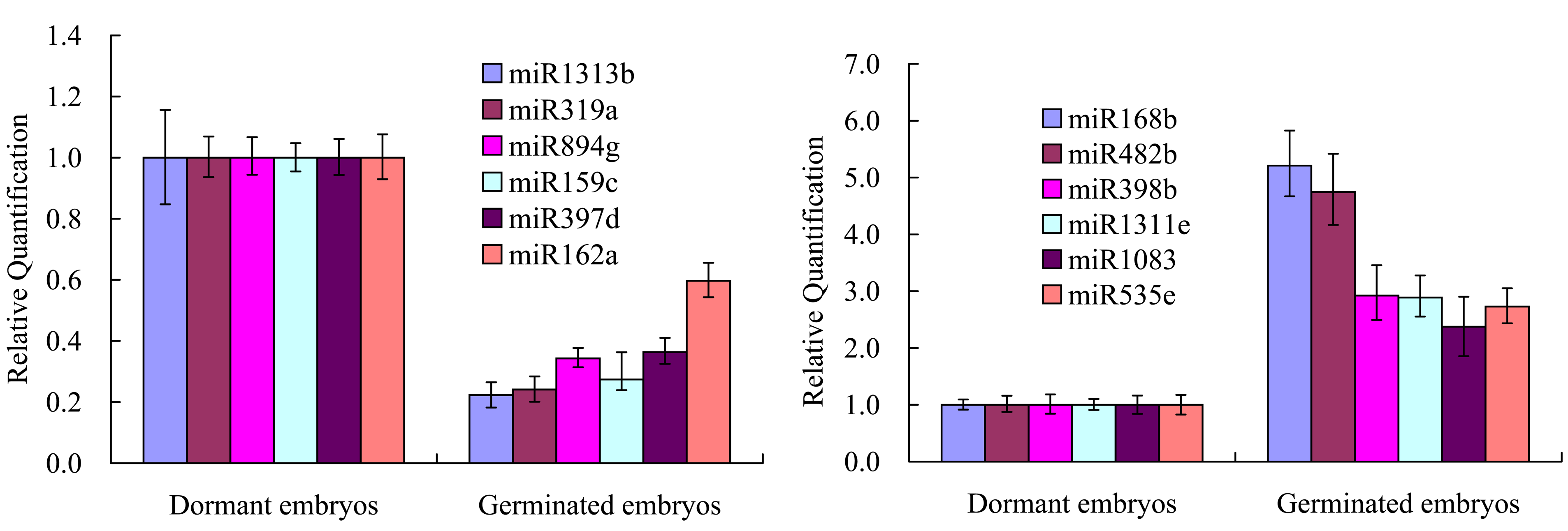

Supplement: Figure S1 — Validation of the miRNA levels of dormant embryos and germinated embryos by qRT-PCR. The results of qRT-PCR validated the sequencing data, albeit with smaller fold-changes between dormant embryos and germinated embryos. (TIF) [file pone.0081452.s001.tif]
