# Supplementary material for: Deciphering Small Noncoding RNAs during the Transition from Dormant Embryo to Germinated Embryo in Larches (Larix leptolepis)
Source: PLoS One. 2013 Dec 10;8(12):e81452. doi: 10.1371/journal.pone.0081452 (PMC3858266; doi:10.1371/journal.pone.0081452)
Supplement: Table S1 — Expression profile of miRNAs between dormant and germinated embryos. (DOC) [file pone.0081452.s002.doc]

**Table S1.** Expression profile of miRNAs between dormant and germinated embryos

| miRNA | Sequence (5’ to 3’) | Dormant embryo  normalized | Germinated embryo normalized | log_2_(dormant/  germinated embryo) |
| --- | --- | --- | --- | --- |
| llemiR-13 | UGAUUCAGGCAUGGAGGAGGACUA | 0.61 | 0.06 | 3.30 |
| miR1313b | UACCGCUGAAAUUAUUGUUCG | 1.71 | 0.19 | 3.20 |
| llemiR-14 | UCUUUCUGAGGCAUGUAUGGGCAU | 0.37 | 0.06 | 2.57 |
| miR319a | UUGGCCUGAAGGGAGCUCCAU | 0.31 | 0.06 | 2.30 |
| miR894g | CGUCUCACGUCGGGUUCACCA | 0.31 | 0.06 | 2.30 |
| miR159c | UUUGGAUUGAAGGGAGCUCUA | 0.24 | 0.06 | 1.98 |
| llemiR-12 | UACACCUCAAGAAAUUGGAUCCCU | 0.24 | 0.06 | 1.98 |
| miR397d | CAUUGAGUGCAGCGUUGAAGA | 0.18 | 0.06 | 1.57 |
| miR162a | UCGAUAAACCUCUGCAUCCAG | 2.45 | 1.18 | 1.05 |
| miR390b | AAGCUCAGGAGGGAUAGCGCU | 0.24 | 0.12 | 0.98 |
| miR162b | UCGAUAAACCUCUGCAUCCGG | 3.61 | 2.11 | 0.78 |
| miR166n | UCGGACCAGGCUUCAUUCUCC | 26.68 | 15.69 | 0.77 |
| miR535d | UGACAACGAGAGAGAGAACGCU | 2.26 | 1.36 | 0.73 |
| miR397a | CAUUGAGUGCAGCGUUGACGA | 92.02 | 55.50 | 0.73 |
| miR159e | UUUGGUUUGAAGGGAGCUUA | 0.98 | 0.62 | 0.66 |
| miR172a | AGAAUCUUGAUGAUGCUGCAU | 0.49 | 0.31 | 0.66 |
| miR947 | UAUCGGAACCUGUUACUGUUUC | 2.69 | 1.80 | 0.58 |
| llemiR-18 | CAACGAUCAACAGGACCACUG | 1.47 | 0.99 | 0.57 |
| miR397b | UAUUGAGUGCAGCGUUGACGA | 153.01 | 105.43 | 0.54 |
| miR171a | UUGAGCCGCGCCAAUAUCACU | 0.98 | 0.68 | 0.52 |
| miR951d | UAUUCUUGACGUCUGGACAACG | 1.10 | 0.81 | 0.45 |
| miR396a | UUCCACAGCUUUCUUGAACUU | 2.45 | 1.86 | 0.40 |
| miR5139b | GGAACCUGGCUCUGAUACCA | 0.24 | 0.19 | 0.40 |
| llemiR-17 | UGAGUCCAGACACACUUCGGCU | 0.24 | 0.19 | 0.40 |
| miR5059a | UCGUGCCUGGGCAGCACCACCA | 18.48 | 14.08 | 0.39 |
| miR950i | UCACAUCUGGCCCACGAUGGUU | 101.74 | 77.70 | 0.39 |
| miR1311c | UCAGAGUUUUGCCGGUUCCCCC | 0.55 | 0.43 | 0.34 |
| llemiR-11 | GCCGUGACCGUGGCGAUCGUGG | 0.55 | 0.43 | 0.34 |
| miR5059d | UCGGACCUGGGCAGCACCACCA | 0.92 | 0.74 | 0.30 |
| miR156a | UGACAGAAGAGAGUGAGCAC | 1074.41 | 888.24 | 0.27 |
| miR167e | UGAAGCUGCCAGCAUGAUCUU | 0.37 | 0.31 | 0.24 |
| miR946d-5P | UGUGGAUACAGAAGGGCUAGU | 0.80 | 0.68 | 0.22 |
| miR5059c | UCGAGCCUGGGCAGCACCACCA | 7.16 | 6.14 | 0.22 |
| miR894e | UGUUUCACGUCGGGUUCACCA | 1.22 | 1.05 | 0.21 |
| miR3701a | UAAACAAUGCCCACCCUUCAUC | 19.33 | 17.12 | 0.18 |
| llemiR-6 | UCCAUGACUUUCCAGAGGGGU | 20.56 | 18.48 | 0.15 |
| llemiR-4 | UUUGAUAGAUCCGAGGUUAAG | 4.28 | 3.97 | 0.11 |
| miR166m | UCGGACCAGGCUUCAUUUCCC | 28.57 | 26.48 | 0.11 |
| miR1311a | UCAGAGUUUUGCCAGUUCCGCC | 0.86 | 0.81 | 0.09 |
| miR5059b | UCGGGCCUGGGCAGCACCACCA | 13.40 | 13.02 | 0.04 |
| miR1312b | UUCGGAGAGAAAAUGGAGACAU | 4.34 | 4.34 | 0.00 |
| miR5139a | CGAAACCUGGCUCUGAUACCA | 0.18 | 0.19 | -0.02 |
| miR5139c | GAAACCUCGCUCUGAUACCA | 0.18 | 0.19 | -0.02 |
| miR951b | UAUUCUUGACGUCUGGACCACG | 525.98 | 547.47 | -0.06 |
| miR1312a | UUCGGAGAGAAAAUGGCGACAU | 2532.36 | 2686.29 | -0.09 |
| miR1314b | UCGGCCUCGAAUGUUAGGAGAU | 6.30 | 6.76 | -0.10 |
| miR166l | UCGGACCAGGCUUUAUUCCCC | 35.49 | 38.14 | -0.10 |
| miR168c | UCGAUUGGUGCAGAUCGGGAA | 3.24 | 3.60 | -0.15 |
| llemiR-2 | UGACCAGUCCUUCUGCGAUCCA | 9.05 | 10.05 | -0.15 |
| miR1314a | UCGGCCUCGAAUGUUAGGAGAA | 231.14 | 258.85 | -0.16 |
| miR894b | CGUUUCACGUCAGGUUCACCA | 1.77 | 2.05 | -0.21 |
| miR167f | UGAAGCUGCUAGCAUGAUCUGG | 0.31 | 0.37 | -0.28 |
| miR167g | UGAAGCUGUCAGCAUGAUCUGG | 0.31 | 0.37 | -0.28 |
| miR1313a | UACCACUGAAAUUAUUGUUCGA | 0.31 | 0.37 | -0.28 |
| miR950d | UCACAUCUGGGCCACGAUGGUU | 3388.89 | 4124.17 | -0.28 |
| miR157 | UUGACAGAAGAUAGAGAGCAC | 5.32 | 6.51 | -0.29 |
| miR894d | CGGUUCACGUCGGGUUCACCA | 1.35 | 1.67 | -0.31 |
| miR894c | CGAUUCACGUCGGGUUCACCA | 5.87 | 7.38 | -0.33 |
| miR950b-3P | UCUGGACCUCGGUGGUUUAUGA | 165.92 | 209.42 | -0.34 |
| miR950c-3P | UAACAUCUGGGCCACGAUGGUU | 22.39 | 28.59 | -0.35 |
| llemiR-8 | UCUGCCUGGUACCUUGACGUA | 6.00 | 7.69 | -0.36 |
| miR950h | CAACAUCUGGGCCACGAUGGUU | 5218.40 | 6954.15 | -0.41 |
| miR169a | CAGCCAAGGAUGACUUGCCGG | 5.69 | 7.63 | -0.42 |
| miR1311d | UCAGAGUUUUGCCAGUUCCACC | 0.37 | 0.50 | -0.43 |
| miR528b | UGGAAGGGGCAUGCAGAGAAG | 0.37 | 0.50 | -0.43 |
| miR946e-5P | UGUGGAUAUAGAAGGGAUAGU | 0.37 | 0.50 | -0.43 |
| miR950a-5p | UCACGUCUGGGCCACGAUGGUU | 30002.53 | 40707.11 | -0.44 |
| miR894a | GUUUCACGUCGGGUUCACCA | 182.63 | 248.31 | -0.44 |
| miR3701b | UAAACAAUUCCCACCCUUCAUC | 10.89 | 14.82 | -0.44 |
| miR482c | UCUUUCCUACUCCUCCCAAGCC | 7.22 | 9.86 | -0.45 |
| miR482a | UCUUCCCUACUCCUCCCAUUCC | 13.64 | 18.98 | -0.48 |
| miR3701c | UAAAUAAUGCCCACCCUUCAUC | 3.43 | 4.78 | -0.48 |
| miR159g | UUUGGUUUGAAGGGAGCUCUU | 0.31 | 0.43 | -0.50 |
| llemiR-10 | UGAGCUCUUGGAAGUGUUGGA | 12.54 | 17.98 | -0.52 |
| miR159f | AUUGGCCUGAAGGGAGCUCCA | 0.43 | 0.62 | -0.53 |
| miR167d | UGAAGCUGCCAGCAUGAUCUGA | 0.86 | 1.24 | -0.53 |
| miR2118b | UUUCCAACGCCUCCCAUGCCUA | 0.86 | 1.24 | -0.53 |
| llemiR-1 | UGCCGUGGUUCGGAGCGAUCGA | 673.30 | 986.34 | -0.55 |
| miR946a-5P | UGUGGAUAUAGAAGGGCUAGU | 541.21 | 805.45 | -0.57 |
| llemiR-7 | UCAUUCCAGUUAUCGUUCUCC | 3.06 | 4.59 | -0.59 |
| miR164b | UGGAGAAGCAGGGCACGUGCU | 0.12 | 0.19 | -0.60 |
| miR5139d | GUAACCUGGCUCUGAUACCA | 0.12 | 0.19 | -0.60 |
| miR5059e | UCGCGCCUGGGCAGCACCACCA | 0.37 | 0.56 | -0.60 |
| miR5072 | UCGUUCCCCAGCGGAGUCGCCA | 0.12 | 0.19 | -0.60 |
| miR166f | UCGGACCAGGGUUCAUUCCCC | 183.79 | 292.46 | -0.67 |
| miR535a | UGACAACGAGAGAGAGCACGCU | 795.36 | 1270.68 | -0.68 |
| miR950e | UGACAUCUGGGCCACGAUGGUU | 8142.81 | 13124.80 | -0.69 |
| miR950j | UCGGGGCCCCUGUGGUUUAUGA | 106.27 | 172.15 | -0.70 |
| llemiR-5 | UGCAAAUGGUGUUUGCGUCGU | 0.98 | 1.61 | -0.72 |
| miR946a-3P | CAGCCCUUCUCCUAUCCACAAU | 2.81 | 4.65 | -0.72 |
| miR950g | UAACGUCUGGGCCACGAUGGUU | 35399.58 | 58560.04 | -0.73 |
| miR397c | UCAUUGGGAGCAGCGUUGAUG | 9.24 | 15.32 | -0.73 |
| miR166j | UCGGACCAGGUUUCAUUCCCC | 59.96 | 100.03 | -0.74 |
| miR319d-5P | AGCUGCCGAAUCAUUCAUUCA | 0.18 | 0.31 | -0.76 |
| llemiR-9 | UCGCAGGUGAGAUGACGCCGGC | 3.18 | 5.46 | -0.78 |
| miR166c | UCUCGGACCAGGCUUCAUUCC | 3310.28 | 5838.44 | -0.82 |
| miR950b-5P | UUACGUCUGGGCCACGAUGGUU | 53793.31 | 94959.92 | -0.82 |
| miR159a | UUUGGUUUGAAGGGAGCUCUA | 18.84 | 34.05 | -0.85 |
| miR159b | CUUGGAUUGAAGGGAGCUCC | 2.32 | 4.22 | -0.86 |
| miR156g | UGACAGAAGAGAGUGGGAAC | 14.81 | 27.04 | -0.87 |
| miR165 | UCGGACCAGGCUUCAUCCCCC | 11.44 | 21.09 | -0.88 |
| miR2862 | UCCAACAGCUCCGAUUCGUCC | 0.49 | 0.93 | -0.93 |
| miR156d | UGACAGAAGAGAGUGGGCAU | 24.35 | 47.81 | -0.97 |
| miR398c | CGUGUUCUCAGGUCGCCCCAG | 0.12 | 0.25 | -1.02 |
| miR398d | CGUGUUCCCAGGUCACCCCAG | 0.06 | 0.12 | -1.02 |
| miR398e | CGUGUUCCCAGGUCGCCCCCG | 0.06 | 0.12 | -1.02 |
| miR482e | UAUUCCCUACUCCUCCCAUUCC | 0.06 | 0.12 | -1.02 |
| miR529c | AGAAGAGAGAGAGCACCGCCU | 0.06 | 0.12 | -1.02 |
| miR166e | UCGGACCAGGAUUCAUUCCCC | 489.51 | 997.57 | -1.03 |
| miR166g | UCGGACCAGGCUUAAUUCCCC | 87.43 | 181.70 | -1.06 |
| miR950c-5P | UGACAUCGGGGCCACGAUGGUU | 68.58 | 145.18 | -1.08 |
| miR390a | AAGCUCAGGAGGGAUAGCGCC | 90.67 | 197.46 | -1.12 |
| miR166a | UCGGACCAGGCUUCAUUCCCC | 21054.27 | 46119.63 | -1.13 |
| miR950f | UUACAUCUGGGCCACGAUGGUU | 179.14 | 393.24 | -1.13 |
| miR168a | UCGCUUGGUGCAGGUCGGGAA | 497.65 | 1103.30 | -1.15 |
| miR167b | UGAAGCUGCCAGCAUGAUCUGG | 14.07 | 32.31 | -1.20 |
| miR2118a | UUCCCUAUUCCUCCCAUUCCUA | 1.53 | 3.53 | -1.21 |
| miR528a | UGGAAGGGGCAUGCAGAGGAG | 2.14 | 4.96 | -1.21 |
| miR398a | UGUGUUCCCAGGUCGCCCCAG | 0.18 | 0.43 | -1.24 |
| miR827 | UUAGAUGAGCAUCAACGAACA | 0.18 | 0.43 | -1.24 |
| miR172b | AGAAUCCUGAUGAUGCUGCAU | 44.23 | 104.87 | -1.25 |
| miR1311b | UCAGAGUUUUGCCGGUUCCACC | 948.98 | 2341.37 | -1.30 |
| miR390c | AAGCUCAGGAGGGAUAGAGCC | 0.12 | 0.31 | -1.34 |
| miR894h | CGUAUCACGUCGGGUUCACCA | 0.12 | 0.31 | -1.34 |
| miR156b | UGACAGAAGAGAGUGGGCAC | 12158.93 | 31263.75 | -1.36 |
| miR946c-5P | UGUGGAUAUGGAAGGGUUAGU | 3.85 | 9.92 | -1.36 |
| miR156f | UGACAGAAGAGAGUGGGCAA | 13.58 | 35.35 | -1.38 |
| miR166b | UCGGACCAGGCUUCAUUCCUC | 997.81 | 2636.43 | -1.40 |
| miR535b | UGACAAAGAGAGAGAGCACGC | 68.03 | 179.90 | -1.40 |
| miR156e | UGACAGAAGAGAGGGGGCAC | 15.85 | 42.11 | -1.41 |
| miR166h | CCGGACCAGGAUUCAUUCCCC | 72.99 | 199.13 | -1.45 |
| miR946b-5P | UGUGGAUACAGAAGGGUUAGGU | 5.02 | 13.95 | -1.48 |
| miR951c | UGUUCUUGACGUCUGGGCCACG | 40.50 | 115.29 | -1.51 |
| miR156c | UGACAGAAGAGAGAGGGCAC | 26.68 | 77.77 | -1.54 |
| miR160 | UGCCUGGCUCCCUGUAUGCCA | 0.12 | 0.37 | -1.60 |
| miR169c | AAGCCAAGGAUGACUUGCCAU | 0.06 | 0.19 | -1.60 |
| miR171b | UUGAGCCGUGCCAAUAUCGCA | 0.06 | 0.19 | -1.60 |
| miR172d | AGAAUCCUGAUGAUGCUGCUU | 0.06 | 0.19 | -1.60 |
| miR393 | UCCAAAGGGAUUGCAUUGAUUC | 0.18 | 0.56 | -1.60 |
| miR166d | CCGGACCAGGCUUCAUUCCCC | 3008.10 | 9172.60 | -1.61 |
| miR167c | CUGAAGCUGCCAGCAUGAUCUG | 0.73 | 2.42 | -1.72 |
| miR529b | AGAAGAGAGAGAGCACUGCC | 98.93 | 326.14 | -1.72 |
| miR167a | UGAAGCUGCCAGCAUGAUCUA | 0.73 | 2.54 | -1.79 |
| miR951a | UGUUCUUGACGUCUGGACCACG | 124.69 | 438.44 | -1.81 |
| miR166i | UCGGACCAGGCUUCCUUCCCC | 70.60 | 255.75 | -1.86 |
| miR319b-5P | AGCUGCCGACUCAUUCAUUCA | 168.31 | 640.24 | -1.93 |
| miR535c | UGACAGAGAGAGAGAGCACGC | 27.53 | 109.02 | -1.99 |
| miR166k | UCGGACCAGGCUUCGUUCCCC | 36.46 | 144.62 | -1.99 |
| miR408 | UGCACUGCCUCUUCCCUGGCU | 0.43 | 1.74 | -2.02 |
| miR319c-5P | AGCUGCCGAUUCAUUCAUUCA | 0.37 | 1.49 | -2.02 |
| miR4414a | UGUGAAUGAUGCGGGAGCUGA | 434.88 | 1769.84 | -2.02 |
| miR535e | UGACAACGAGAGAGAGCACGCC | 2.75 | 13.71 | -2.32 |
| miR4414c | UGUGAAUGAUGCGGGAGCUGU | 3.98 | 21.89 | -2.46 |
| miR1083 | UAGCCUGGAACGAAGCACGC | 17.62 | 98.73 | -2.49 |
| miR1311e | UCAGAGUUUUGCCGGUUCCGCC | 0.06 | 0.37 | -2.60 |
| miR4414e | UGUGAAUGAUGCGGGAGAUGA | 0.12 | 0.74 | -2.60 |
| llemiR-16 | UCGGAAUGCUGGAGGAGGCAA | 0.06 | 0.37 | -2.60 |
| miR164a | UGGAGAAGCAGGGCACGUGCG | 23.86 | 167.94 | -2.82 |
| miR398b | CGUGUUCCAAGGUCGCCCCAG | 0.06 | 0.50 | -3.02 |
| miR482b | UCUUUCCUACUCCUCCCAUUCC | 0.06 | 0.62 | -3.34 |
| miR529 | AGAAGAGAGAGAGCACUGCU | 0.06 | 0.62 | -3.34 |
| miR529a | UGAAGAGAGAGAGCACAGCCC | 4.71 | 83.91 | -4.15 |
| miR4414b | UGUGAACGAUGCGGGAGCUGA | 6.79 | 250.79 | -5.21 |
| miR169b | GAGCCAAGGAUGAUUUGCCGA | 0.06 | 2.60 | -5.41 |
| miR168b | UCGCUUGGUGCAGAUCGGGAC | 0.06 | 6.08 | -6.63 |
| miR159d | UUUGGAUUGAAGGGAGCUCUG | 0.24 | 0 | - |
| miR396c | UUCCACGGCUUUCUUGAACUU | 0.12 | 0 | - |
| miR894f | CGUUUCACGUCGGGUUCAUCA | 0.37 | 0 | - |
| llemiR-3 | UCAAGUGUUUCUGGACUCACC | 0.31 | 0 | - |
| miR164c | UGGAGAAGCAGGGCACGUGAG | 0 | 0.37 | - |
| miR164d | UGGAGAAGCAGGGAACGUGCG | 0 | 0.31 | - |
| miR164e | UGGAGAAGCAGGGCACGUGCA | 0 | 0.25 | - |
| miR166o | UCGGACCAGGCUUCUUUCCCC | 0 | 71.50 | - |
| miR172c | AGAAUCCUGAUGAUGAUGCAU | 0 | 0.31 | - |
| miR396b | UUCCACAGCUUUCUUGAACUA | 0 | 0.25 | - |
| miR399a | CGCCAAAGGAGAGUUGCCCUG | 0 | 0.31 | - |
| miR399b | UGCCAAAGGAGAGUUGCCCUG | 0 | 0.12 | - |
| miR482d | UCUUCCCUAAACCUCCCAAACC | 0 | 0.06 | - |
| miR536 | UCGUGCCAAGCUGUGUGCAUC | 0 | 0.43 | - |
| llemiR-15 | UCAGUGAGCUUAGGGUACGUUGG | 0 | 0.25 | - |
| llemiR-19 | UGUGACGGGGAUGGGAUGCU | 0 | 0.68 | - |
| miR4414d | UGUGAAUGAUGAGGGAGCUGA | 0 | 1.74 | - |
