# Supplementary material for: Deciphering Small Noncoding RNAs during the Transition from Dormant Embryo to Germinated Embryo in Larches (Larix leptolepis)
Source: PLoS One. 2013 Dec 10;8(12):e81452. doi: 10.1371/journal.pone.0081452 (PMC3858266; doi:10.1371/journal.pone.0081452)
Supplement: Table S2 — miRNA primers used for qRT-PCR in larch. (DOC) [file pone.0081452.s003.doc]

Table S2. miRNA primers used for qRT-PCR in larch.

NCode™ universal qPCR primer (Invitrogen) was used as reverse primer.

| miRNA | Forward primer (5′→ 3′) | Length | Tm |
| --- | --- | --- | --- |
| lle-miR1313b | GTACCACTGAAATTATTGTTCGAAA | 25 | 58.0 |
| lle-miR319a | GGACTGAAGGGAGCTCCCTAA | 21 | 60.0 |
| lle-miR894 | GTTTCACGTCGGGTTCACCAAA | 22 | 64.4 |
| lle-miR159c | CTTGGATTGAAGGGAGCTCCA | 21 | 61.6 |
| lle-miR397d | ATTGAGTGCAGCGTTGACGAA | 21 | 61.6 |
| lle-miR162 | TCGATAAACCTCTGCATCCAGAA | 23 | 61.5 |
| lle-miR168b | GCTTGGTGCAGATCGGGACAA | 21 | 65.4 |
| lle-miR482b | TTCCCTATTCCTCCCATTCCTAA | 23 | 61.2 |
| lle-miR398b | TGTTCCCAGGTCGCCCCAGAA | 21 | 69.1 |
| lle-miR1311 | TCAGAGTTTTGCCAGTTCCGCC | 22 | 66.2 |
| lle-miR1083 | GCCTGGAACGAAGCACGCAAA | 21 | 67.9 |
| lle-miR535 | ACAACGAGAGAGAGCACGCTAAA | 23 | 61.3 |
| lle-miR159a | GTTTGGTTTGAAGGGAGCTCTAA | 23 | 59.9 |
| lle-miR160a | CTGGCTCCCTGTATGCCAAA | 20 | 60.9 |
| lle-miR166a | GACCAGGCTTCATTCCCCAA | 20 | 62.0 |
| lle-miR397a | TTGAGTGCAGCGTTGACGAA | 20 | 60.6 |
| lle-miR398a | GTGTTCCCAGGTCGCCCCAGAA | 22 | 67.0 |
