# Supplementary material for: Deciphering Small Noncoding RNAs during the Transition from Dormant Embryo to Germinated Embryo in Larches (Larix leptolepis)
Source: PLoS One. 2013 Dec 10;8(12):e81452. doi: 10.1371/journal.pone.0081452 (PMC3858266; doi:10.1371/journal.pone.0081452)
Supplement: Table S3 — Primers for qRT-PCR analysis of target genes. (DOC) [file pone.0081452.s004.doc]

Table S3. Primers for qRT-PCR analysis of target genes.

| **Target genes** | **Gene specific forward primer**  **5’ to 3’** | **Gene specific reverse primer**  **5’ to 3’** |
| --- | --- | --- |
| JR164246 (*DCL1*) | *DCL1*-1:AGGTTAGATGTGAAAATGTGGGG | *DCL1*-2:CTGAAGAGAAGCCTTGGTGAT |
| JX416702 (*DCL3*) | *DCL3*-1:TTTCAGTTGGTAGAACAAGAGCG | *DCL3*-2:AAGTTTTTCTCTATCAGGAATGTGC |
| JR153061(*RDR2*) | *RDR2*-1: GCAAACACCAAGATTTACGATGA | *RDR2*-2: AACATTGACAGCATGACAAAACTC |
| JR164402(*RDR6*) | *RDR6*-1: TGGAAACTTCTGGATTTGA GGA | *RDR6*-2: TGATTTCGCCCTGCTTTCTG |
| JX157847(*MYB33*) | QT159-1: TGGGAGTTTGATGATTGG | QT159-2: AAGTTGGCTTGAAGGGAG |
| JR145514(*ARF*) | QT160-1:TGGGAGGACTTCAAATGGCTA | QT160-2:GATTGGAGTTTGTTCGGATGA |
| JR158006(*HD-ZIPⅢ*) | QT166-1:CAGAGGAGACTTTGACGCAG | QT166-2:CAGAACATCCAAGCACCGACA |
| JR147962(*Laccases*) | QT397-1:GCTGTAAAACAAGGAGAAACG | QT397-2:GTCTTGTATGGTTTGGTGTAGCA |
| JR143920(*plastocyanin*) | QT398-1:GGTGTTAGGGTTTGGTGTTGG | QT398-2:ATGGTCCATCCTGAGCTTCC |
